# Significant impact of different oxygen breathing conditions on noninvasive *in vivo *tumor-hypoxia imaging using [^18^F]-fluoro-azomycinarabino-furanoside ([^18^F]FAZA)

**DOI:** 10.1186/1748-717X-6-165

**Published:** 2011-11-25

**Authors:** Florian C Maier, Manfred Kneilling, Gerald Reischl, Funda Cay, Daniel Bukala, Andreas Schmid, Martin S Judenhofer, Martin Röcken, Hans-Jürgen Machulla, Bernd J Pichler

**Affiliations:** 1 Department of Preclinical Imaging and Radiopharmacy, Laboratory for Preclinical Imaging and Imaging Technology of the Werner Siemens-Foundation, University of Tübingen, Germany; 2 Department of Dermatology, University of Tübingen, Germany

## Abstract

**Background:**

[^18^F]FAZA is a PET biomarker with great potential for imaging tumor hypoxia. Aim of our study was to compare [^18^F]FAZA uptake in mice with subcutaneous exogenous CT26 colon carcinomas and endogenous polyoma middle-T (PyV-mT) mammary carcinomas and to analyze the influence of different breathing protocols in CT26 colon carcinomas as well as the reversibility or irreversibility of [^18^F]FAZA uptake.

**Methods:**

We injected subcutaneous CT26 colon carcinoma or polyomavirus middle-T (PyV-mT) mammary carcinoma-bearing mice intravenously with^18^F-FAZA and performed PET scans 1-3 h post injection (*p.i.*). To analyze the impact of oxygen supply in CT26 carcinomas we used three different breathing protocols: (P0) air; (P1) 100% oxygen 1 h prior injection until 3 h *p.i.*; (P2) 100% oxygen breathing starting 2 min prior tracer injection until 1 h *p.i. *and during the PET scans; mice were breathing air between the 2 h and 3 h 10 min static scans. Normalized PET images were analyzed by using defined regions of interest. Finally, some mice were dissected for pimonidazole immunohistochemistry.

**Results:**

There was no difference in^18^F-FAZA uptake 1-3 h *p.i. *between the two carcinoma types (CT26: 1.58 ± 0.45%ID/cc; PyV-mT: 1.47 ± 0.89%ID/cc, 1 h *p.i.*, tumor size < 0.5 cm^3^). We measured a significant tracer clearance, which was more pronounced in muscle tissue (P0). The [^18^F]FAZA tumor-to-muscle-ratios in CT26 colon carcinoma-bearing mice 2 h and 3 h, but not 1 h *p.i. *were significantly higher when the mice breathed air (P0: 3.56 ± 0.55, 3 h) compared to the oxygen breathing protocols (P1: 2.45 ± 0.58; P2: 2.77 ± 0.42, 3 h). Surprisingly, the breathing protocols P1 and P2 showed no significant differences in T/M ratios, thus indicating that the crucial [^18^F]FAZA uptake phase is during the first hour after [^18^F]FAZA injection. Importantly, the muscle clearance was not affected by the different oxygen breathing conditions while the tumor clearance was lower when mice were breathing air.

**Conclusion:**

Exogenous CT26 colon carcinomas and endogenous polyoma middle-T (PyV-mT) mammary carcinomas showed no differences in [^18^F]FAZA uptake 1-3 h *p.i. *Our analysis using various breathing protocols with air (P0) and with pure oxygen (P1, P2) clearly indicate that [^18^F]FAZA is an appropriate PET biomarker for *in vivo *analysis of hypoxia revealing an enhanced tracer uptake in tumors with reduced oxygen supply. [^18^F]FAZA uptake was independent of tumor-type.

## Introduction

Tumor hypoxia, one of the major hallmarks of malignant tumor disease, is indicative of more aggressive tumor progression and is associated with angiogenesis and metastasis [1-4]. Hypoxic tumor tissue is also known to be more resistant to radiotherapy and chemotherapy compared to normally oxygenated tumor tissue [1]. Identifying and targeting the hypoxic areas of tumors is pivotal for selecting patients who require additional or more specific treatment strategies. Several invasive and noninvasive techniques are currently available, although oxygen electrode systems for detecting hypoxia are not clinically used due to their invasiveness and limitations concerning the accessibility of the tumor [5,6]. However, noninvasive *in vivo *identification and quantification of hypoxic tumor regions using positron emission tomography (PET) is a field of growing interest and has been investigated in recent years [7,8]. Currently, a variety of PET tracers are available for hypoxia imaging, such as [^18^F]fluoromisonidazole ([^18^F]FMISO) [9-13], [^18^F]FAZA [14-16], [^124^I]-iodo-azomycinarabino-furanoside ([^124^I]IAZA) [17,18] and [^64^Cu]-(II)diacetyl-bis(N4-methylthiosemicarbazone) ([^64^Cu]ATSM) [19-21]. The 2-Nitroimidazole tracers show selective uptake in hypoxic cells both *in vitro *and *in vivo*. In contrast [^64^Cu]ATSM binding may also involve mechanisms independent of hypoxia [22]. In line with this Yuan *et al. *have recently reported that [^64^Cu]ATSM is a valid hypoxia marker for some tumors but not for all as enhanced tracer uptake was found also in areas without any hypoxia but with enhanced perfusion [23].

However, for reliable use of 2-Nitroimidazole tracers in clinical settings, further evaluation studies are needed. [^18^F]FAZA and [^18^F]FMISO are currently two of the PET biomarkers with great promise for imaging tumor hypoxia [24] and have been the focus of clinical studies. 2-Nitroimidazole tracers such as [^18^F]FAZA are readily diffusible through cell membranes and undergo reversible reduction by intracellular reductases to yield nitroimidazole radical anions, which are then rapidly reoxidized to neutral molecules that diffuse out of the cell. If the concentration of oxygen in tumor tissue is low, oxidation competes with the irreversible binding of the radical anion to other substrates, which forms irreversible compounds with cellular macromolecular components. Therefore, with decreasing intracellular concentration of oxygen the tracer accumulates within the hypoxic tissue. Piert et al. proved the principle of the particular trapping mechanism in a porcine hypoxic liver model by applying extensive *in vivo *and *ex vivo *analyses that revealed a direct influence of the oxygen concentration on tracer accumulation [9,25]. An alternative approach to proving the dependence of hypoxia tracer accumulation on the oxygen concentration is the change of breathing air or oxygen by employing different breathing protocols. The inverse correlation between [^18^F]FMISO and [^18^F]FAZA uptake and tumor tissue oxygenation has been shown in our previous studies [14]. According to Piert et al., [^18^F]FAZA does not show intertumoral differences in total tumor uptake as evaluated by biodistribution in three different xenograft mouse tumor-models [14]. However, there has not been a study that has investigated the dependency of tracer uptake on different breathing protocols with various oxygen incubation times. Thus, in the present study, we focused on the further evaluation of [^18^F]FAZA using an exogenous and an endogenous tumor model with three different breathing protocols and compared the results with *in vivo *[^18^F]FDG-scans and *ex vivo *pimonidazole immunohistochemical analyses of the tumors. Importantly, because clinical observations in patients suggest a reversible binding of [^18^F]FAZA [26], we also focused on this particular issue in a preclinical experiment and analyzed the reversibility of [^18^F]FAZA binding in hypoxic tumor tissue and normoxic muscle tissue using different oxygen breathing protocols.

## Materials and methods

### Tracer synthesis

For a detailed description of all tracer synthesis please see Additional file 1.

### Animal models

C57BL/6 mice expressing the polyomavirus middle T (PyV-mT) antigen under the control of the mammary-specific mouse mammary tumor virus long terminal repeat promoter (MMTV-LTR) [27,28], were kindly provided by Lesley G Ellies, from the School of Medicine at the University of California at San Diego (La Jolla, USA) and were bred at the animal care facilities at the University of Tübingen. For imaging experiments, transgenic PyV-mT C57BL/6 mice were grown to 18 weeks of age. At this age, the spontaneous mammary tumors reached a volume of 0.982 ± 0.958 cm^3 ^(n = 20). On average, each mouse had 5 tumors along the mammary glands [27,28].

BALB/c mice were purchased from Charles River Laboratories and were between 8 and 10 weeks of age. CT26 mouse colon carcinoma cells were kindly provided by Prof. Dr. med. Ralph Mocicat (Institute of molecular immunology, German Research Center for Environmental Health, Munich, Germany) and analyzed for mycoplasma infection once per month. For CT26 mouse colon carcinoma cell culture please see Additional file 1. CT26 cells (10^6 ^in 200 μL phosphate-buffered saline; PBS [29,30]) were subcutaneously injected in the right shoulder of female BALB/c mice. Thirteen days after tumor inoculation, the tumors reached a volume of 0.279 ± 0.086 cm^3 ^(n = 10).

All animal experiments were performed according to the current guidelines for the care and use of research animals under the German Animal Protection Law. Animal experiments were approved by the local government (Regierungspräsidium Tübingen).

### *In vivo* PET imaging studies

PET studies investigating the tumor type-dependent uptake of [^18^F]FAZA were conducted with PyV-mT C57/BL6 mice (n = 9 mice, n = 20 tumors) and CT26 tumor-bearing BALB/c mice (n = 10 mice, n = 10 tumors). Under isoflurane anesthesia, the tail veins of the mice were injected with 14.2 ± 0.5 MBq [^18^F]FDG or 12.3 ± 1.0 MBq [^18^F]FAZA on two consecutive days. [^18^F]FDG scans were performed to identify large necrotic tumor regions in mice with large PyV-mT mammary carcinomas (> 1.5 cm^3^) that were subsequently ignored during [^18^F]FAZA PET image analysis. In addition we performed [^18^F]FDG PET scans in some CT26 colon carcinoma bearing mice and could not detect large necrotic areas. For [^18^F]FDG, a 1 h uptake time was allowed prior to a 10 min static scan. Mice were kept under 1.5% isoflurane anesthesia with an air flow rate of 0.6 L/min using a dedicated vaporizer (Vetland, Louisville, KY, USA) for the entire uptake and scan time. The body temperature of mice was maintained at 37°C on the scanner bed and in the incubation chambers by using heating pads. During the [^18^F]FAZA uptake time, mice were kept conscious in room air. The main study was performed using static PET scan protocols described in the next section (breathing protocols). However, prior to onset of our study we performed antecedent dynamic studies to ensure that the static PET scans are performed during the transient [^18^F]FAZA-equilibrium phase. Static 10 min duration PET-scans were performed at 1 h, 2 h and 3 h *p.i. *using an Inveon-dedicated PET scanner (Siemens Healthcare, Knoxville, TN, USA) with an axial field of view of 12.7 cm and a spatial resolution of 1.4 mm (full width at half maximum, FWHM) [31]. After the last [^18^F]FAZA PET scan, the animals were sacrificed and the tumors were harvested for *ex vivo *analysis.

### Breathing protocols

To prove the principle of hypoxia detection using [^18^F]FAZA, we investigated the specific uptake in CT26 colon carcinoma-bearing BALB/c mice when the mice breathed normal air with 21% (P0)) or 100% oxygen (P1); we expected a lower [^18^F]FAZA uptake when breathing 100% oxygen as a consequence of an enhanced systemic oxygen supply. To obtain more information about the importance of air or 100% oxygen breathing on [^18^F]FAZA uptake during the preincubation time and the first hour after [^18^F]FAZA administration, we designed a third protocol in which mice breathed 100% oxygen 2 minutes prior and 1 h after [^18^F]FAZA administration (P2). Moreover, we analyzed the role of air breathing in between the PET scans (P1 vs. P2).

**Figure 1:**
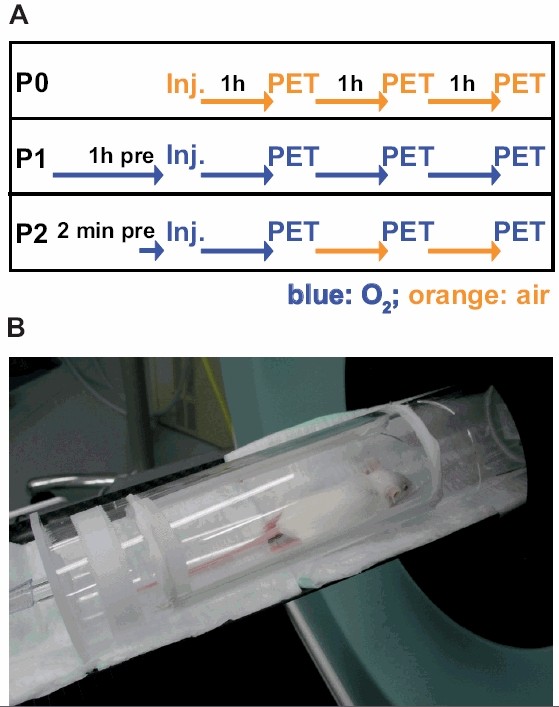
**Breathing protocols with increasing oxygen incubation times**. A, to analyze the impact of oxygen supply in CT26 carcinomas we used three different breathing protocols: (P0) air; (P1) 100% oxygen 1 h prior injection until 3 h *p.i.*; (P2) 100% oxygen breathing starting 2 min prior tracer injection until 1 h *p.i. *and during the PET scans; mice were breathing air between the 2 h and 3 h 10 min static scans. The flow chart indicates the injection time points (Inj.) and the PET scans (PET) relative to the uptake times and oxygen or air breathing protocols. B, custom-made chamber for the microPET scans. Animals were anesthetized with 1.5% isoflurane and 0.6 L/min carrier gas flow during injection and microPET scans (either air or 100% oxygen, depending on the breathing protocol).

Breathing protocols were as follows (Figure 1A):

• P0: room air during the entire procedure (n = 10; 21% oxygen);

• P1: 100% oxygen 1 h prior to tracer injection and during the entire scan time (n = 7);

• P2: 100% oxygen 2 min prior to tracer injection, 1 h *p.i. *and during the scans, which were performed at 2 h and 3 h *p.i. *The mice breathed room air between the scans (n = 7).

Mice were kept in a chamber under 100% oxygen at a flow rate of 0.6 L/min without anesthesia during the pre-injection time (P1, P2) and during the uptake phase after the injection. During the scans, the animals were placed in a custom-made chamber on the scanner bed (Figure 1B). Mice were kept conscious except during tracer injection (1 min) and PET scans (10 min). Again, [^18^F]FDG scans were preemptively performed to confirm the viability of the tumor tissue. We divided the CT26 colon carcinoma-bearing mice into two groups as CT26 colon carcinomas reach an exponential growth phase at day 12 to 14 after CT26 cell inoculation which, in turn, would cause a higher heterogeneity of the tumors, as the larger CT26 tumors showed a tendency toward higher [^18^F]FAZA uptake (Table 1). The first group (n = 4) was scanned under the P0 protocol and on the subsequent day under the P1 protocol; the second group (n = 3) was scanned under the P0 and P2 protocols. In addition, we scanned mice individually using the breathing protocols P0 (n = 6), P1 (n = 3) and P2 (n = 4) to obtain at least seven mice per breathing protocol group (P0, P1 and P2) for analysis.

**Table 1:** Tumor type and size-dependency of [^18^F]FAZA in CT26 colon carcinomas and PyV-mT mammary carcinomas

Tumor	Tumor size	1 h *p.i.*	2 h *p.i.*	3 h *p.i.*
PyV-mT (n = 20)	0.982 ± 0.958 cm^3^	1.31 ± 0.71	1.06 ± 0.69	0.92 ± 0.61
CT26 (n = 10)	0.279 ± 0.086 cm^3^	1.53 ± 0.43	1.14 ± 0.37	0.84 ± 0.26
PyV-mT (n = 12)	0.15 - 0.50 cm^3^	1.47 ± 0.89	1.13 ± 0.83	0.97 ± 0.70
PyV-mT (n = 4)	0.5 - 1.5 cm^3^	1.22 ± 0.59	1.05 ± 0.61	0.91 ± 0.56
PyV-mT (n = 4)	> 1.5 cm^3^	1.15 ± 0.41	0.86 ± 0.36	0.76 ± 0.42
CT26 (n = 4)	< 0.15 cm^3^	1.37 ± 0.41	0.92 ± 0.38	0.68 ± 0.38
CT26 (n = 6)	0.15 - 0.50 cm^3^	1.58 ± 0.45	1.22 ± 0.35	0.90 ± 0.22

Data are presented as %ID/cc ± SD. h: hour, *p.i.*: post injection. PyV-mT n = 9 mice, n = 20 tumors.

### PET data analysis

PET data were reconstructed using an OSEM 2D algorithm into a 128 × 128 image matrix (resulting in a final voxel size of 0.79 × 0.79 × 0.8 mm^3^). After correction for decay, the data were analyzed by drawing standardized volumes of interest (VOIs). In the transverse image planes, a circular region of interest (ROI) of 4.8 mm in diameter was placed over the maximum activity in the tumor, and two ROIs of 3.2 mm in diameter were placed over the slices directly above and below the slice of maximum activity. In this way, it could be ensured that regions outside the tumors as well as large necrotic areas in PyV-mT carcinomas > 1.5 cm^3 ^were not included in the analysis VOI. Furthermore, we expected a "dilution effect" if all tumor regions - hypoxic as well as non-hypoxic areas - were included in the analysis. The shoulder muscle (biceps) of the left forelimb was used as a reference region. The size of the muscle was confirmed by a mouse anatomy atlas [32]. [^18^F]FDG scans supported the VOI definition, as the dimension of the muscle was clearly visible. Two ROIs of 5 mm in diameter were placed over two slices in the muscle. Image analysis was performed with the microPET ASIPro VM software package (Siemens Healthcare, Knoxville, TN, USA). The slice with the highest radioactivity concentration in the tumor was identified visually and with the navigation function of the software, which identified the pixel with the highest intensity. Quantitative PET data were reported as the percentage of the injected dose per cm^3 ^(%ID/cc) or as the tumor-to-muscle ratio (T/M).

### Histology and immunohistochemistry

For the assessment of tumor hypoxia, pimonidazole (60 mg/kg, Chemicon International, Temecula, CA, USA) was injected in some CT26 colon carcinoma bearing mice intraperitoneally 1 h before the mice were sacrificed. Tumors were fixed in 4% formalin, embedded in paraffin and cut into 5 μm slices with a manual rotary microtome (RM2235; Leica, Nussloch, Germany). Slices were analyzed using the Hypoxyprobe™-1 Kit for the detection of tissue hypoxia (Chemicon International, Temecula, CA, USA) with a confocal microscope (DM 5000B, Leica, Wetzlar, Germany) and FITC-fluorescence (primary antibody: Hypoxyprobe-1Mab1). A control stain was performed with PBS instead of the primary antibody. Hematoxylin and eosin (H&E) stains were performed on subsequent slices according to standard procedures [33].

### Statistics

Differences in [^18^F]FAZA tumor type-dependent uptake were compared using a two-sample Student's t-test. Differences in [^18^F]FAZA uptake according to the breathing protocol were analyzed using the Wilcoxon test and the JMP software package (SAS Institute Inc, Cary, NC, USA). Data were represented as the mean ± standard deviation (SD). A value for p < 0.05 was considered to be statistically significant, and a value for p < 0.01 was highly statistically significant.

## Results

### Evaluation of tumor type-dependent [^18^F]FAZA uptake

**Figure 2:**
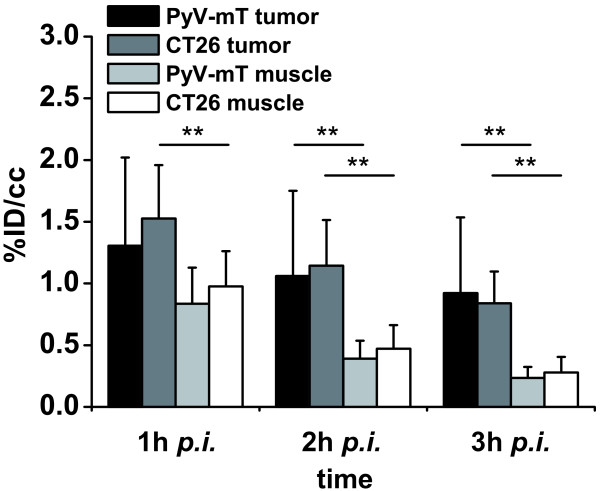
**[^18^F]FAZA tumor and muscle uptake in both tumor models**. The [^18^F]FAZA muscle clearance is clearly more pronounced compared to tumor clearance (** p < 0.01; PyV-mT C57/BL6, n = 9; CT26 colon carcinoma-bearing BALB/c, n = 10). Note that there is no difference in between the two animal models in terms of tracer clearance from both tumor and muscle tissue (breathing protocol P0, air).

**Figure 3:**
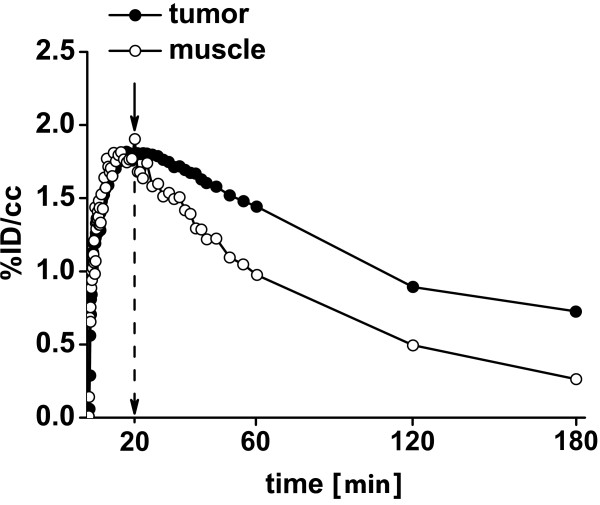
**Dynamic [^18^F]FAZA-PET scan**. [^18^F]FAZA-uptake in a CT26 colon carcinoma over 3 h using breathing protocol P0 (see also Figure 1A). Note that the peak uptake is achieved 20 minutes *p.i.*

PyV-mT mammary carcinoma and CT26 colon carcinoma-bearing mice were imaged 1 h, 2 h and 3 h after injection of 12.3 ± 1.0 MBq [^18^F]FAZA using breathing protocol P0 (Figure 1A and 1B; P0 breathing protocol). Absolute tracer uptake decreased in the PyV-mT mammary carcinomas from 1.31 ± 0.71 %ID/cc at 1 h to 0.92 ± 0.61 %ID/cc at 3 h and decreased in the CT26 colon carcinomas from 1.53 ± 0.43 %ID/cc at 1 h to 0.84 ± 0.26 %ID/cc at 3 h (Figure 2, Table 1). Absolute tracer uptake in muscle tissue in both mouse strains (C57BL/6, BALB/c) decreased much more pronounced than in carcinomas. No statistically significant differences in [^18^F]FAZA uptake were found between the endogenous PyV-mT mammary carcinomas and the exogenous subcutaneous CT26 colon carcinomas (p = 0.29; Figure 2, Table 1). Figure 3 represents the dynamics of the [^18^F]FAZA uptake in a CT26 colon carcinoma-bearing mouse over 3 h (breathing protocol P0), indicating that the peak uptake was achieved 20 min post injection (*p.i.*), whereas the T/M is highest at 3 h *p.i.*

The [^18^F]FAZA-PET data from PyV-mT mammary carcinomas and CT26 colon carcinomas were further analyzed according to tumor sizes (Table 1). Endogenous PyV-mT mammary carcinomas were classified into three groups: 1) tumors that were smaller than 0.5 cm^3^; 2) tumors that were between 0.5 cm^3 ^and 1.5 cm^3 ^and 3) tumors that were larger than 1.5 cm^3^. Statistical analysis did not reveal any significant differences at any scan time between small, medium sized, and large PyV-mT mammary carcinomas. Similar to the PyV-mT mammary carcinomas, the CT26 colon carcinomas were classified into tumor lesions that were smaller than 0.15 cm^3 ^and tumor lesions that were between 0.15 cm^3 ^and 0.5 cm^3^. Similar to the PyV-mT mammary carcinomas, we did not observe any significant differences in [^18^F]FAZA uptake between the small and medium sized CT26 colon carcinomas. However, in the CT26 tumor model, there was a tendency toward a higher tumor uptake with increasing tumor size, which is in contrast to the PyV-mT model in which the opposite effect was observed (Table 1).

**Figure 4:**
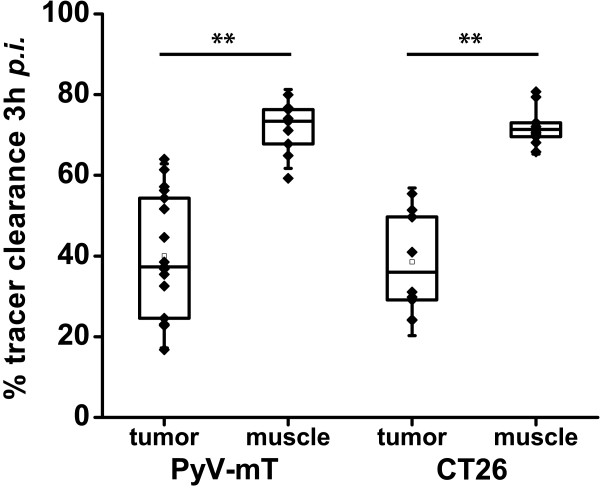
**[^18^F]FAZA tumor and muscle clearance in both tumor models**. [^18^F]FAZA clearance is displayed in both animal models (relative to 1 h after injection) with a higher muscle clearance (** p < 0.01; PyV-mT C57/BL6, n = 9; CT26 colon carcinoma-bearing BALB/c, n = 10).

Investigations of the [^18^F]FAZA clearance revealed a significant relative clearance of 40 ± 15% in the endogenous PyV-mT mammary carcinomas (mice: n = 9, tumors: n = 20) and a similar significant relative clearance of 39 ± 12% in the exogenous CT26 colon carcinomas (n = 10) 3 h after tracer injection (p < 0.01 compared to %ID/cc 1 h after injection, Figure 4). The [^18^F]FAZA clearance was significantly higher in the muscle tissue of the PyV-mT mammary carcinoma (73 ± 6%; C57BL/6) and the CT26 colon carcinoma-bearing mice (72 ± 5%; BALB/c) compared to the mammary and colon carcinomas (Figure 4, p < 0.01). Importantly, both tumor types showed a similar tracer clearance (p = 0.90). The rapid clearance from the non-hypoxic muscle tissue compared to the hypoxic tumor tissue explains the improved detectability of hypoxic tumor tissue at later scan time points (Figure 2, Figure 4).

**Figure 5:**
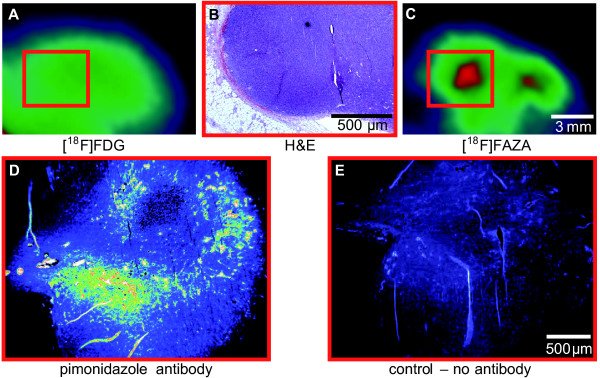
**Comparison of *in vivo *[^18^F]FAZA- and [^18^F]FDG images and pimonidazole immunohistochemistry of the same tumor**. A and C, Typical sample of [^18^F]FDG- and [^18^F]FAZA-PET images of a CT26 colon carcinoma (tumor size category: 0.15-0.5 cm^3^). B, *ex vivo *analysis of the same CT26 colon carcinoma (A and C) by H&E staining of tumor slices. Red squares in A and C indicate the H&E-stained tumor region. D and E, *ex vivo *pimonidazole immunohistochemistry of the areas indicated in A and C. The mice were injected with 60 mg/kg pimonidazole 1 h prior to *ex vivo *analysis. Tumor slices were analyzed using the Hypoxyprobe™-1 kit for the detection of tissue hypoxia. Control staining was performed with PBS instead of the primary antibody.

To exclude necrotic areas in the CT26 colon carcinomas, we also performed [^18^F]FDG scans on some of the mice. Importantly, we did not identify any impaired [^18^F]FDG uptake in the CT26 colon carcinomas, which is a sign of necrosis (Figure 5A). This was further confirmed by H&E staining of the slices of the CT26 colon carcinomas, which displayed no signs of necrotic tumor tissue (Figure 5B). The correlation between *in vivo *[^18^F]FAZA scans (Figure 5C) and *ex vivo *pimonidazole immunohistochemistry staining of the hypoxic areas in slices of CT26 colon carcinomas (Figure 5D) further confirmed the specificity of [^18^F]FAZA to hypoxic tumor tissue.

### Oxygen incubation time-dependent [^18^F]FAZA tumor uptake

**Figure 6:**
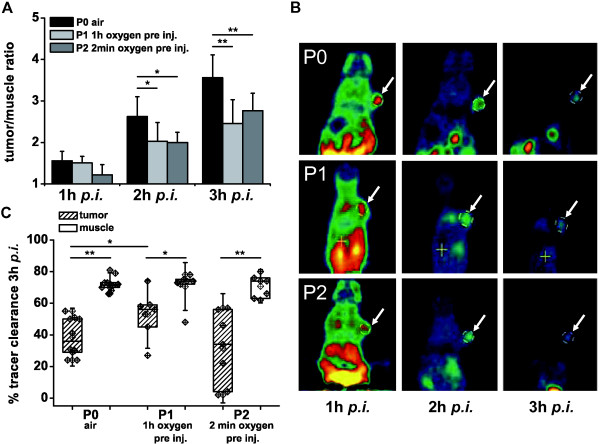
**Assessment of [^18^F]FAZA tumor-to-muscle ratios in CT26 colon carcinomas under different breathing protocols**. A, we used three different breathing protocols: (P0) air; (P1) 100% oxygen 1 h prior injection until 3 h *p.i.*; (P2) 100% oxygen breathing starting 2 min prior tracer injection until 1 h *p.i. *and during the PET scans; mice were breathing air between the 2 h and 3 h 10 min static scans. B, an example of microPET images of CT26 colon carcinoma-bearing mice (arrowheads indicate tumors). Images were taken 1 h, 2 h and 3 h after injection of [^18^F]FAZA following the P0, P1 and P2 protocols. C, [^18^F]FAZA clearance (relative to 1 h after injection) from tumor and muscle tissue with a clear oxygen dependency (* p < 0.05; ** p < 0.01; P0: n = 10, P1: n = 7, P2: n = 7).

We analyzed the role of oxygen breathing during the preincubation time (2 min vs. 1 h) and during the first hour after [^18^F]FAZA-injection using three different breathing protocols with different air or 100% oxygen incubation times (Figure 1A) in the CT26 colon carcinoma-bearing mice. Moreover, we analyzed the role of air breathing between the 1 h, 2 h, and 3 h PET scans (P1, P2). During the scans, the animals were placed in a custom-made chamber on the scanner bed (Figure 1B). Tumor-to-muscle ratios of [^18^F]FAZA 2 h and 3 h *p.i. *were significantly higher (p < 0.01) when the mice breathed air (3.56 ± 0.55; P0) compared to the oxygen breathing protocols P1 (2.45 ± 0.58; 100% oxygen 1 h prior tracer injection and during the entire scan time) and P2 (2.77 ± 0.42; partly 100% oxygen breathing; Figure 6A), proving the principle of hypoxia detection. Interestingly, we observed no differences in tumor-to-muscle ratios 1 h after [^18^F]FAZA-injection when the mice breathed room air (P0) or 100% oxygen (P1). Moreover, we did not detect any significant differences in the tumor-to-muscle ratios between the oxygen breathing protocols P1 and P2 (Figure 6A). Because the mice breathed 100% oxygen 60 minutes after [^18^F]FAZA- injection (until the first PET scan) in both protocols (P1 and P2), it seems that the duration of 100% oxygen breathing (2 min vs. 1 h) prior to the [^18^F]FAZA- injection does not significantly influence the results for both early and late PET scans. Figure 6B displays representative [^18^F]FAZA-PET images of the CT26 colon carcinoma-bearing mice under the P0, P1, and P2 breathing protocols 1 h, 2 h, and 3 h after [^18^F]FAZA-injection. Interestingly, the muscle clearance was unaffected by the different oxygen breathing conditions and remained at 72 ± 5% for P0, 71 ± 10% for P1 and 71 ± 7% for P2 (Figure 6C). However, the rate of tumor clearance was significantly lower when the mice breathed room air (P0; 39 ± 12%) compared to when they breathed 4 h of pure oxygen (P1; 53 ± 14%; p < 0.05). In contrast, the P2 breathing protocol resulted in a very heterogeneous tumor [^18^F]FAZA clearance of 32 ± 23% (Figure 6C).

## Discussion

A variety of PET tracers for noninvasive tumor hypoxia imaging are currently available [9-21,24-26]. The lack of sufficient *in vivo *evaluation makes judgments about the predictive value of these traces in the detection of hypoxic tumor regions difficult. However, various reports have demonstrated that [^18^F]FAZA, compared to [^18^F]FMISO, is a valid hypoxia imaging agent [14-16]. Furthermore, [^18^F]FAZA has been shown to be superior in comparison to [^124^I]IAZA. In a study by Reischl *et al. *[^124^I]IAZA displayed no improvement in tumor to muscle ratios although the tracer uptake could be followed for up to 48 h after injection. This could be either related to a loss of ^124^I or a hint for reversibility of the uptake mechanism [17]. Piert *et al. *further reported that [^18^F]FAZA did not show intertumoral differences in terms of total tumor uptake in 3 different xenograft mouse tumor models [14]. Nevertheless, there was a lack of endogenous tumor models in that study, and the above-mentioned conclusions were only based on post mortem biodistribution results. We have chosen these two tumor-types to study an endogenous (PyV-mT mammary carcinoma) and an exogenous carcinoma model (CT26 colon carcinoma) to identify potential differences between the uptake patterns in exogenous and endogenous carcinomas. Our study reveals that [^18^F]FAZA displays no difference in uptake between the exogenous (CT26) and the endogenous (PyV-mT) mouse tumor model. Furthermore, there was no connection between uptake and tumor size when using [^18^F]FAZA although our data show that there is a tendency toward higher uptake in larger tumors in the CT26 model, whereas we observed the opposite effect in the PyV-mT model (Table 1). Small lesions were difficult to detect in PyV-mT mice along the mammary glands, whereas small tumors (< 0.15 cm^3^) were clearly identifiable in the CT26 mice. Lipophilicity and plasma half-life are fundamental attributes that influence tumor-to-background ratios and total uptake. [^18^F]FAZA has a high potential for rapid diffusion into hypoxic tissue and fast clearance from non-hypoxic tissue compared to other hypoxia tracers, such as [^18^F]FMISO [14]. [^18^F]FAZA indeed displayed a substantial clearance from the tumor and muscle tissue, with a significantly faster and more substantial clearance from the muscle, which provides more evidence that the trapping in hypoxic cells is reversible (Figure 2). Busk *et al. *showed in a mouse study that hypoxic squamous cell carcinomas of the cervix display a washout type [^18^F]FAZA-TAC [34]. The authors hypothesize that the observed washout could be due to the rapid tracer clearance in mice, which may be even more critical for the hypoxic to non-hypoxic tumor tissue image contrast than the rate of specific binding [34]. However, there are also *in vitro *as well as clinical data suggesting that compounds incorporating a 2-nitroimidazole as functional unit are characterized by a reversible uptake in tumors [26,35]. Moreover Busk *et al. *showed an evident loss of [^18^F]FAZA in different tumor cell lines *in vitro *[35], while Shi *et al. *recommend in a study focusing on patients with head and neck cancer to favour a reversible 2-compartmental model [26]. Consequently the reversible uptake in carcinomas represents a major drawback of [^18^F]FAZA as the image contrast is affected by the perfusion and the vascular architecture.

In our study, we found that the clearance from muscle tissue was unaffected by the different breathing protocols, whereas the tumor clearance showed a tendency toward higher clearances with longer oxygen incubation times (Figure 6). This contrasts with the findings from Reischl *et al. *who reported that the reduced T/M ratio under carbogen (95% oxygen, 5% CO_2_) breathing was related to a reduced muscle washout whereas the tumor clearance remained unchanged [17]. On the other hand, Mortensen *et al. *observed in mammary carcinoma bearing carbogen breathing mice primarily lower [^18^F]FAZA tumor to blood ratios compared to air breathing mice [36].

Furthermore, the muscle tissue was not affected by the different breathing protocols, as was the case for the tumor tissue; this also illustrate the specificity of [^18^F]FAZA for hypoxic tumor tissue. A potential benefit of nitroimidazoles such as [^18^F]FAZA might be the underlying bioreductive mechanism in hypoxic cells. [^18^F]FAZA is mainly reduced by cytosolic xanthine oxidase upon entering the hypoxic cell and is only reduced to a small extent by the mitochondrial respiratory chain complex I [20]. Thus, it follows that the reduction of [^18^F]FAZA is not dependent upon NADH levels (or the "redox-state" of the cell) but is directly linked to the intracellular oxygen level. This is also true for other nitroimidazoles.

To our knowledge, this is the first study to correlate [^18^F]FAZA tumor uptake with different oxygen breathing protocols (P0, P1, P2) to determine the cell oxygen level-dependent uptake of [^18^F]FAZA as other investigators (Piert *et al. *and Reischl *et al*.) analyzed exclusively littermates that breathed air or 95% oxygen with 5% CO_2 _[14]. The tumor-to-muscle ratios of [^18^F]FAZA at 2 h and 3 h *p.i. *were higher using the P0 breathing protocol (21% oxygen) compared to the P1 and P2 oxygen breathing protocols. [^18^F]FAZA tumor uptake showed an inverse correlation with oxygen supply, which confirms the specificity for hypoxia detection (Figure 6). Surprisingly, the P0 breathing protocol and the P1 and P2 oxygen breathing protocols did not show significant differences in the T/M ratios 1 h post [^18^F]FAZA-injection.

Subsequently, the similar tracer uptake in tumors in the P1 and P2 breathing protocols during the investigations 2-3 h *p.i. *clearly indicate that the crucial uptake phase for [^18^F]FAZA occurs during the first hour after injection because air breathing in between the 1 h, 2 h and 3 h PET scans (P2) did not significantly impair [^18^F]FAZA uptake compared to the oxygen breathing protocol (P1). Sequential [^18^F]FDG (Figure 5A) and [^18^F]FAZA (Figure 5C) scans followed by *ex vivo *H&E (Figure 5B) and pimonidazole immunostaining (Figure 5D and 5E) strongly support the feasibility of [^18^F]FAZA in terms of tumor hypoxia imaging.

## Conclusion

We could not detect any difference in [^18^F]FAZA uptake regarding the two tumor types and the tumor size. The uptake and clearance of [^18^F]FAZA in carcinomas was dependent on the oxygen supply, especially during the time prior to and until 1 h after [^18^F]FAZA-injection, whereas the uptake and clearance of [^18^F]FAZA in muscle tissue was not affected by different oxygen breathing protocols. The different oxygen breathing protocols clearly indicate that [^18^F]FAZA is a valid marker for hypoxia because higher tracer uptake in tumors correlates with decreased oxygen supply. The critical time for [^18^F]FAZA uptake is the first hour after tracer injection, whereas the pre-injection incubation time has little or no effect on the hypoxia-specific tracer uptake in tumor tissue. Thus, different oxygen breathing protocols clearly confirmed that [^18^F]FAZA is an appropriate PET biomarker for *in vivo *analysis of hypoxia, especially for late measurements.

## Competing interests

Our laboratory is the European reference and training site of Siemens Preclinical Solutions.

## Authors' contributions

FCM and MK planned and performed all *in vivo* and *ex vivo* studies, analyzed the data, drafted the manuscript and carried out the statistical analysis. GR developed and synthesized PET-tracers and helped planning the studies. FC helped planning and performing *in vivo *and *ex vivo *studies. DB carried out CT26 cell culture, injected CT26 subcutaneous colon carcinomas and typecasted PyV-mT mice. AS helped evaluating the data and drafting the manuscript. MSJ helped planning the studies, drafting the manuscript and evaluating the data. MR helped drafting the manuscript. HJM developed tracers and edited the manuscript. BJP helped designing and coordinating all studies, drafted and edited the manuscript and reviewed the data. All authors read and approved the final manuscript.

## Supplementary Material

Additional file 1 **Tracer synthesis and cell culture conditions of CT26 mouse colon carcinoma cells**. In additional file 1, we have listed a detailed description of all radiotracer synthesis ([^18^F]FAZA and [^18^F]FDG) along with the exact cell culture conditions of CT26 mouse colon carcinoma cells. Click here for file
