# Supplementary material for: Significant impact of different oxygen breathing conditions on noninvasive in vivo tumor-hypoxia imaging using [18F]-fluoro-azomycinarabino-furanoside ([18F]FAZA)
Source: Radiat Oncol. 2011 Nov 25;6:165. doi: 10.1186/1748-717X-6-165 (PMC3283532; doi:10.1186/1748-717X-6-165)
Supplement: Additional file 1 — Tracer synthesis and cell culture conditions of CT26 mouse colon carcinoma cells. In additional file 1, we have listed a detailed description of all radiotracer synthesis ([18F]FAZA and [18F]FDG) along with the exact cell culture conditions of CT26 mouse colon carcinoma cells. [file 1748-717X-6-165-S1.DOC]

*Tracer synthesis*

Fluorine-18 was produced as [^18^F]-fluoride at the PETtrace cyclotron (General Electric Healthcare, Uppsala, Sweden) using [^18^O]-H_2_O (Rotem, Israel) and the ^18^O(p,n)^18^F nuclear reaction. [^18^F]FDG was synthesized in a FDG MicroLAB (GE Healthcare) according to Hamacher *et al*. [27] using mannose triflate (ABX, Radeberg, Germany) as a precursor. Radiochemical purity as determined by TLC was > 95 %. [^18^F]FAZA was synthesized as previously described [28; 29]. Briefly, azeotropically dried [^18^F]-fluoride was reacted in a TRACERlab FX_F-N_ synthesis module (GE Healthcare) with 5 mg of 1-(2,3-di-*O*-acetyl-5-*O*-tosyl-α-*D*-arabinofuranosyl)-2-nitroimidazole (ABX) in 1 mL DMSO (100 °C; 5 min) followed by hydrolysis with 1 mL of 0.1 N NaOH for 2 min at room temperature. After neutralization and preparative HPLC, the product solution was filter sterilized. Radiochemical yields were 21 ± 4% (10 ± 2 GBq), the radiochemical purity was > 95%, and the specific activity was > 50 GBq/µmol.

*Cell culture conditions of CT26 mouse colon carcinoma cells*

CT26 mouse colon carcinoma cells were cultured in Dulbecco's modified Eagle medium (DMEM) containing 9% fetal calf serum (FCS; PAA, Cölbe, Germany), 40 µM 2-mercaptoethanol (Sigma, Steinheim, Germany), 50 U penicillin/streptomycin (Biochrom AG, Berlin, Germany), 9 mM HEPES (Biochrom AG, Berlin, Germany) and 1 mM sodium pyruvate (Gibco, Eggenstein, Germany) supplemented with 1% 50x MEM amino acids (Gibco) at 37 °C in a humidified atmosphere with 5% CO_2_.
